# Spatial Variations and Determinants of Acute Malnutrition Among Under-Five Children in Ethiopia: Evidence from 2019 Ethiopian Demographic Health Survey

**DOI:** 10.5334/aogh.3500

**Published:** 2021-11-22

**Authors:** Binyam Tariku Seboka, Tilahun Dessie Alene, Habtamu Setegn Ngusie, Samuel Hailegebreal, Delelegn Emwodew Yehualashet, Girma Gilano, Mohammedjud Hassen Ahmed, Robel Hussen Kabthymer, Girum Gebremeskel Kanno, Getanew Aschalew Tesfa

**Affiliations:** 1 School of public health, Dilla University, Dilla, Ethiopia; 2 Department of Pediatrics and Child Health, College of Medicine and Health Sciences, Wollo University, Dessie, Ethiopia; 3 Department of Health informatics, College of Health Sciences, Mettu University, Mettu, Ethiopia; 4 Department of Health Informatics, Arbaminch University, Arbaminch, Ethiopia

## Abstract

**Background and aims:**

Childhood acute malnutrition, in the form of wasting defined by a severe weight loss as a result of acute food shortage and/or illness. It is a critical public health problem that needs urgent attention in developing countries, like Ethiopia. Despite its variation between localities, the risk factors and its geospatial variation were not addressed enough across the various corner of the country. Therefore, the current study was undertaken to assess spatial variation and factors associated with acute malnutrition among under-five children in Ethiopia.

**Methods:**

A total weighted sample of 4 955 under-five children were included from the 2019 Demographic and Health Survey. Getis-Ord spatial statistical tool used to identify the hot and cold spot areas of severe and acute malnutrition. A multilevel multivariable logistic regression model using was used to examine predictors of acute malnutrition. In the multivariable multilevel analysis, Adjusted Odds Ratio with 95% CI was used to declare significant determinants of acute malnutrition among children.

**Result:**

Among 4 955 under-five children, 7% of them were wasted and 1% of them were severely wasted in Ethiopia during the 2019 national demographic survey. The distribution was followed some spatial geo-locations where most parts of Somali were severely affected (RR = 1.46, P37 value <0.001), and the distribution affected few areas of Afar, Gambella, and Benishangul Gumz regions. Factors that significantly associated with childhood wasting were: gender(male)1.9 (1.3–2.7), age (above 36 months) 0.5 (0.2–0.9), wealth index(richest) 0.5 (0.2–0.8), and water source (unimproved source) 1.5 (1.0–2.3).

**Conclusions:**

Our finding implies, the distribution of childhood wasting was not random. Regions like Afar, Somali, and pocket areas in Gambella and SNNP should be considered as priority areas nutritional interventions for reducing acute malnutrition. The established socio-demographic and economic characteristics can be also used to develop strategies.

## Introduction

Childhood malnutrition is well estimated as the major underlying risk factor for morbidity and mortality in children under five years [1,2]. Acute malnutrition also known as wasting is characterized by a rapid deterioration in nutritional status over a short period that causes a child to become too thin for his or her height because of weight loss or failure to gain weight [3,4,5]. For children, it can be measured using the weight-for-height nutritional index or mid-upper arm circumference [6,7]. It is defined as moderate acute malnutrition (MAM) and severe acute malnutrition (SAM) whereas; MAM: is WHZ≥ –3Z score &<–2Z score or MUAC ≥ 115 mm & < 125 mm (≥11.5 cm & < 12.5 cm) and SAM: is defined by visible severe wasting, or by the presence of bilateral pitting edema of nutritional origin, or WHZ< –3Z score or MUAC <115 mm (<11.5 cm) in children aged 6–59 months [2].

Globally, between 8 to 11 million under-five children die each year [8]. More than 35% of these deaths are attributed to undernutrition and 1 in 12 children (8%, 52 million) were wasted [9]. It is also one of the major causes of childhood deaths in developing countries [10,11,12]. More than 90% of undernourished people live in developing countries [2]. Africa carries the heaviest burden of under-nutrition [9], in which a recent study indicated that 39.9 % of under-five children group affected by malnutrition [13], and the prevalence of wasting in East Africa is 6% [14].

Ethiopia has adopted a multi-sectorial nutrition policy and has been implementing nutrition programs with some success [15,16]. In this regard, Ethiopia design the program called “the sustainable under-nutrition reduction in Ethiopia (SURE)” which is a government-led multi-sector intervention that helps to integrate the work of the health and agriculture sectors to deliver a complex multicomponent intervention to improve child feeding, diversified diet, and nutritional behavioral modification to reduce under-nutrition [16]. However, under-nutrition remains high and suffers from a very high burden of acute and chronic malnutrition [17], with almost half of Ethiopian children chronically malnourished and 1 in 10 children wasted [18]. According to the Ethiopian Demographic and Health Survey (EDHS) quick list of, 2005, 2011, 2016, and 2019 the prevalence of under-five wasting was 12.2%, 9.7%, 9.9%, and 7.0% respectively [18,19,20,21].

Multiple factors contribute to childhood wasting. The common determinants reported by several studies include gender, age of the child [7,22,23,24], monthly income [22,24], diarrhea in the previous two weeks [25,26,27], not consuming additional food during pregnancy/lactation [28,29], non-exclusive breastfeeding practices [26,29], larger family size [26], mothers education [22,26], presence of ARI [26,31], attending ANC [7,29,32], immunization status [33,32,31,30,29,28,27,26,25,24], mother not having consumed extra food during this pregnancy/lactation [4,22].

Most of the previous studies conducted in Ethiopia were not examine the extent of the variation within and between regional wasting in Ethiopia and the variation of predictors across regions. Moreover, the previous studies conducted in Ethiopia used binary logistic regression which leads to biased results. The assumptions of independence among individuals within the same clusters and of equal variance across clusters are violated in the case of grouped data [35]. Hence, a multi-level analysis, which has a number of advantages over binary logistic regression, is the appropriate statistical analysis method for such a study. The main concerns of the authors in this study were to identify the spatial distribution and associated factors of wasting aged 6 to 59 months in Ethiopia using spatial multilevel analysis.

## Methods and Materials

### Study design and Setting

We have used the Ethiopian Demographic Health Information Survey (EDHS) of 2019 to identify factors associated with wasting which were community-based cross-sectional surveys conducted across the country. Ethiopia, the most populous country in Africa, is situated in the Horn of Africa between 3 and 15 degrees north latitude and 33 and 48 degrees east longitude (3°–15° N and 33°–48°E). It has an administrative structure of nine regional states (Tigray, Afar, Amhara, Oromiya, Somali, Benishangul-Gumuz, Southern Nations Nationalities and People [SNNP], Gambela, and Harari) and two city administrations (Addis Ababa and Dire Dawa). These are subdivided into 68 zones, 817 administrative districts which are further divided into 16 253 Kebeles, the smallest administrative units of the country. It has an estimated population of 114.96 million in 2020, which makes it second in Africa and 12th in the world’s most populous country.

### Data source, Extraction, Sampling Procedure, and Study Participants

Our data source was the EDHS survey which was collected in 2019. EDHS is collected every five years by the Ethiopian Central Statistical Agency (CSA) along with ICF International and funded by USAID. The data sets for EDHSs were downloaded in SPSS format with permission from the Measure DHS website (*http://www.dhsprogram.com*). The shapefile of the map of Ethiopia has been accessed as an open-source without restriction from Open Africa website (*https://africaopendata.org/dataset/ethiopia-shapefiles*).

The EDHSs samples were collected using stratified in a two-stage cluster sampling technique. In the first stage, each region was stratified into urban and rural areas. In the second stage of selection, a fixed number of households per cluster were selected with an equal probability of systematic selection from the newly created household listing. All women age 15–49, who were either permanent residents of the selected households or visitors who slept in the household the night before the survey, were eligible to be interviewed. All under-five children within five years during the surveys in Ethiopia were the source of the population for this study, whereas all under-five children in the selected enumeration areas (EAs) within five years during the survey were the study population. Ultimately, a total representative sample of 5 057 under-five children was included in the 2019 survey [18,20,21].

Geographic coordinates of each survey cluster were also collected using Global Positioning System (GPS) receivers. To ensure confidentiality, GPS latitude/longitude positions for all surveys were randomly displaced before public release. The detailed procedure has been presented in each EDHSs report [18,20,21].

### Variables of study

#### Outcome variable

In this study, the dependent variable was under-five wasting which is defined as the percentage of under-five children whose weight-for-height z-score (WHZ) is below –2 SD in the national center for health statistics (NCHS) growth curve. Therefore, we consider under-five wasting (wasted = 1 or not wasted = 0) as the outcome variable [14].

Yij = \left\{ {\begin{array}{*{20}{c}}
{1,\;\;\;\;\;\;|\;if\ there\ is\ wasting}\\
{0\ if\ there\ is\ no\ wasting}
\end{array}} \right.

When Y is the outcome variable (wasting), while i is for the individual-level factors, while j is for the community factors.

#### Independent Variables

The independent variables included: socio-demographic and economic factors: age, sex, occupation, educational status, head of household, wealth index, and religion, geographical factors (region, residence, and temperature), maternal health service utilization factors (antenatal care, place of delivery, and postnatal care), nutritional status of mother (BMI and HFA), birth weight, the timing of breastfeeding, clinical factors (anemic status of the mother, anemic status of the child), drinking safe water, latrine use and media exposure of respondents. Early initiation of breastfeeding –infants who are sucking the breast milk within one hour of birth. Introduction of solid, semi-solid, or soft foods (6–8 months), birth interval were factors for childhood wasting [3,4,7,24,25,26,30,32,36].

### Data Management and Analysis

After downloading EDHS data, sample weights were applied to compensate for the unequal probability of selection between each stratum, data cleaning and recording were carried out in SPSS statistical software version 24. The EDHS datasets were joined to Global Positioning System (GPS) coordinates of EDHS using the joining variable as recommended by DHS measure.

#### Spatial Analysis

The data was exported into Arc GIS 10.8 to visualize key estimation, clusters, and regional variation among wasting. For the spatial analysis, ArcGIS version 10.8 and Sat Scan version 9.6 statistical software were used for exploring the spatial distribution, global spatial autocorrelation, spatial interpolation, and for identifying significance. The spatial autocorrelation (Global Moran’s I) statistic measure was used to evaluate whether the spatial distribution of wasting was random or not. Moran’s I is a spatial statistic used to measure spatial autocorrelation by taking the entire data set and produce a single value that ranges from –1 to + 1. Moran’s I values close to –1, 1, and 0 indicate wasting was dispersed, wasting was clustered, and wasting was distributed randomly, respectively. A statistically significant Moran’s I (P < 0:05) leads to rejection of the null hypothesis (wasting is randomly distributed) and indicates the presence of spatial autocorrelation.

The local Getis-Ord G index (LGi) was used to analyze causality autocorrelation into positive and negative. If the prevalence rates had similar attributes of high or low values (high-high or low-low autocorrelation), they were defined as positive autocorrelation whereas if the attributes had opposing values (high-low or low-high autocorrelation) they were defined as negative autocorrelation. Moreover, the spatial interpolation technique was applied to predict the un-sampled/unmeasured value from sampled measurements.

Autocorrelation can be classified into positive and negative correlations through the local Getis-Ord G positive autocorrelation occurs when similar values are clustered together on a map (high rates surrounded by nearby high rates or low rates surrounded by nearby low rates). Negative autocorrelation indicates different values clustered together on a map, that is, high values surrounded by nearby low values or low values surrounded by nearby high values. Statistical significance of autocorrelation was determined by z-scores and p-value with a 95% level of confidence. The distribution and variations of wasting prevalence rates among children across the country were displayed on the map.

Using Kuldorff’s SaTScan version 9.6 program, spatial scan statistical analysis was used to classify statistically important hotspot areas. To fit the Bernoulli model, we used wasting under-five children as cases and not wasted children as controls. The numbers of cases in each location have Bernoulli distribution and a maximum spatial cluster size of < 50% of the population was used as an upper limit. Z-score is computed to determine the statistical significance of clustering, and the *P*-value was used to determine if the number of observed 6 to 59 months aged children who were within the potential cluster was significant or not. The null hypothesis of no clusters was rejected when the *P*-value ≤ 0.05. Based on 999 Monte Carlo replications the significant clusters were identified and ranked based on their likelihood ratio test [37,38].

#### Statistical Analysis

The multivariable multilevel logistic regression model was used to determine the effect of different factors on wasting. For this multilevel analysis, four models were constructed. Those are the null model without predictors (Model I), model II with only individual-level variables, model III with only community-level variables, and model IV both individual-level and community-level variables. For model comparison, we used the log-likelihood ratio (LLR) and deviance. The highest log-likelihood or the smallest deviance wins the best-fitted model. Therefore, model III which includes both individual and community-level variables was selected as the best fit model for the data.

An adjusted OR (AOR) with 95% CIs was computed to identify the independent factors of under-five wasting at p value<0.05. A multicollinearity test was done in order to rule out a significant correlation between variables. If the values of variance inflation factor (VIF) were lower than 10, then the collinearity problem was considered less likely. Correlation coefficient (ICC), a proportional change in community variance (PCV), and median odds ratio (MOR) were used for measuring variation or random effect [35].

The intra-class correlation coefficient is a measure of within-cluster variation (i.e. the variation between individuals within the same cluster). The PCV is a measurement of the total variation attributed to individual and/or community-level factors at each model. The MOR is the median odds ratio between the individual of higher propensity and the individual of lower propensity when comparing two individuals from two different randomly chosen clusters and it measures the unexplained cluster heterogeneity (the variation between clusters) by comparing two persons from two randomly chosen different clusters. The MOR measure is always greater than or equal to “1.” If The MOR measure is “1,” there is no variation between clusters. The within-cluster correlation was measured using intra-cluster correlation (ICC) which is expected to be 10% to use the model. The ICC, PCV, and MOR were determined using the estimated variance of clusters using the following formula:

ICC = \frac{V}{{V + {\rm{\pi }}2/3}}, Where V is a variance of estimated clusters, {\rm{MOR}} = Exp\sqrt {2 \times {\rm{V}} \times 0.6745}

PCV = \frac{{\left({{\rm{VA}} - {\rm{VB}}} \right)}}{{V{\rm{B}}}} \times 100, were VA = variance of the initial model; VB = variance of the model with more terms.

The multilevel analysis model is one of the analysis methods that can correctly handle the correlated data. A multilevel model evaluates how factors at different levels affect the dependent variable. A multilevel model provides correct parameter estimates by correcting the biases introduced from clustering by producing correct SEs, thus producing correct CI and significance tests.

### Ethical Consideration

Publicly available EDHSs data were used for this study. Ethical approval of EDHS was obtained from the ICF Institutional Review Board (IRB), Ethiopia Health and Nutrition Research Institute Review Board, and the Ministry of Science and Technology. For this particular study, a brief description of the protocol was submitted to the MEASURE DHS program to access and analyze the data. Permission was obtained from the program to access and analyze the data. During EDHS data collection, Informed consent was taken from each participant, and all identifiers were removed and the confidentiality of the information was maintained.

## Result

### Socio-Demographic Characteristics

***Table 1*** reports selected socio-demographic and economic characteristics of the included participants. A total weighted sample of 4 955 children aged 6–59 months with their mothers was included in this study. Of the total children, 2 008 (40.5%) were in the age range of 36–59 months. The majority of the children, 2 517 (50.1) were males. Regarding region, 2 017 (39.9%), 1 016 (20.1%), and 964 (19.1%) were from Oromia, Southern Nation Nationalities and Peoples Region (SNNPR), and Amhara respectively. Of the total, 3 787 (75.0%) lived in the rural areas and 1 170 (23.6%) were from the poorest households (***Table 1***).

**Table 1:** The Descriptive Characteristics of the Study Participants.


VARIABLES	WASTED	NOT WASTED		WASTED	NOT WASTED

	WEIGHTED FREQUENCY (%)	WEIGHTED FREQUENCY (%)	VARIABLES	WEIGHTED FREQUENCY (%)	WEIGHTED FREQUENCY (%)

**Sex of child**			**Wealth index**		

Male	223.8 (62.7)	2293.6 (49.9)	Poorest	135.5 (37.9)	1034 (22.5)

Female	132.9 (37.3)	2305.0 (50.1)	Poorer	80.0 (22.4)	1002.4 (21.8)

**Age of child**			Middle	47.1 (13.2)	875.7 (19.0)

0–5m	48.6 (13.6)	467.9 (10.2)	Richer	55.4 (15.5)	809.8 (17.6)

6–11m	29.3 (8.2)	437.3 (9.5)	Richest	38.7 (10.9)	876.4 (19.1)

12–23m	78.8 (22.1)	911.3 (19.8)	**Child birth order**		

24–35m	76.3 (21.4)	897.8 (19.5)	First	113.6 (31.8)	1816.6 (39.5)

>36m	123.6 (34.6)	1884.4 (41)	Second	80.2 (22.5)	1216.2 (26.5)

**ANC visit**			Third	162.9 (45.7)	1565.9 (34.1)

Yes	161.2 (65.1)	2524.7 (75.8)	**Mother educational level**		

No	86.3 (34.9)	803.8 (24.2)	No education	247.7 (69.5)	2426 (52.8)

**Under-five children in house**			Primary	86.0 (24.1)	1655 (36.0)

1 child	103.8 (29.2)	1836.4 (40.1)	Secondary& above	122.9 (6.4)	516.5 (11.2)

2 child	180.0 (50.6)	2124.7 (46.4)	**Household size**		

3 child	72.2 (20.3)	621.7 (13.6)	1–4	70.7 (19.8)	1333.4 (29.0)

**Source of drinking water**			5–9	248.6 (69.7)	2951.5 (64.2)

unimproved	129.9 (36.4)	1626.1 (35.4)	10 and more	37.4 (10.5)	313.7 (6.8)

Improved	226.8 (63.6)	2969.4 (64.6)	**Residence**		

**Vaccination status**			urban	70.4 (19.7)	1161.9 (25.3)

Yes	63.4 (17.5)	825.7 (17.9)	Rural	286.3 (80.3)	3436.7 (74.7)

No	294.4 (82.5)	3772.8 (82.0)			


### Spatial Distribution and Clustering of Wasted Children In Ethiopia

**Figure 1:**
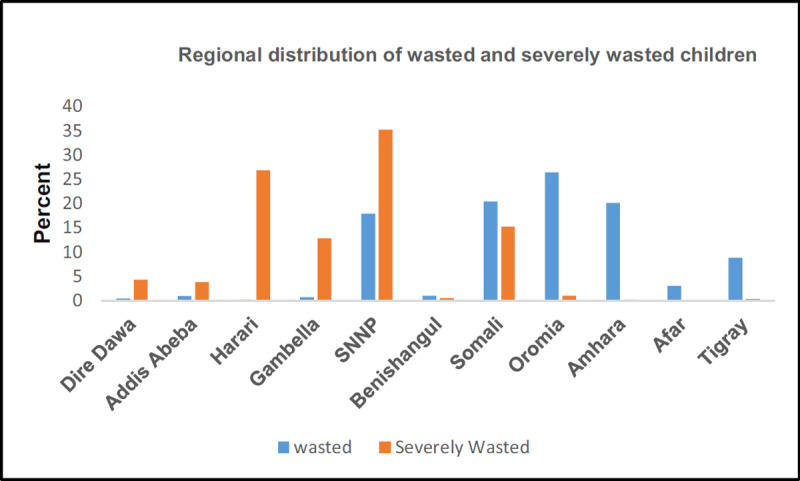
Acute and severe malnutrition among children aged 6–59 months in Ethiopia, 2019 EDHS.

***Figure 1*** shows the distribution of acute and severe malnutrition regionally among under-five children in Ethiopia. The prevalence of childhood wasting shows a variation across regions; in 2019, the range was from 26.4% in the Oromia region to 0.2% in the Harari region. However, the highest distribution of both acute and severe malnutrition was found in the SNNP and Somali regions.

**Figure 2:**
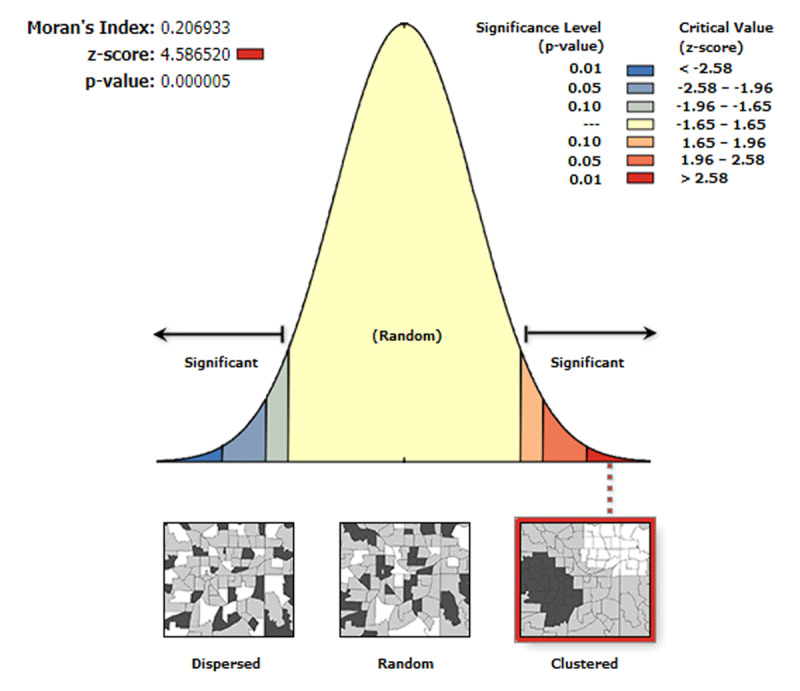
The global spatial autocorrelation of wasted children in Ethiopia, 2019 EDHS.

**Figure 3:**
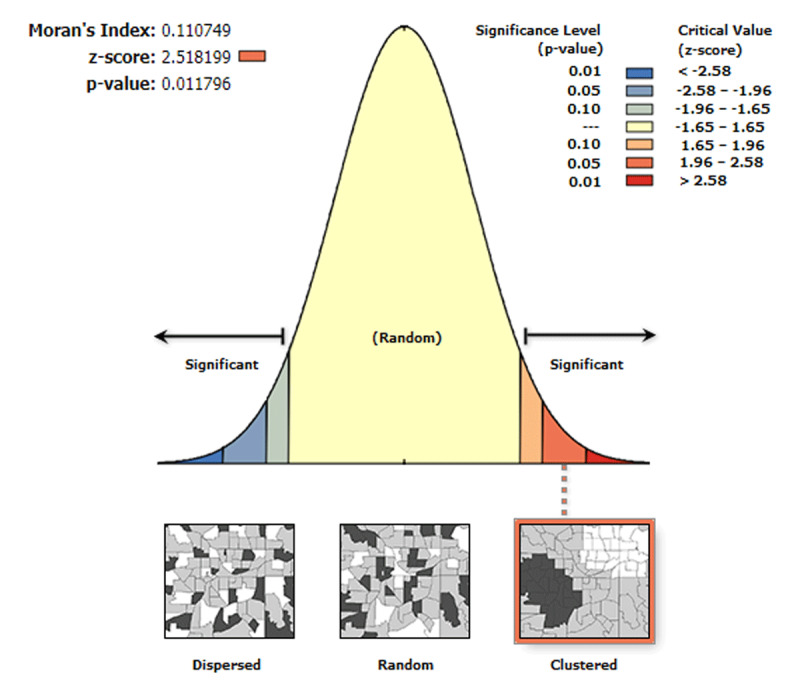
The global spatial autocorrelation of severely wasted children in Ethiopia, 2019 EDHS.

The spatial distribution of wasted and severely wasted children varied across regions in Ethiopia. Results of the Global Moran’s I values (0.21 and 0.11) indicated that there was significant clustering of wasted and severely wasted children, respectively. Besides, the Z-scores of 4.58 and 2.52, respectively, also indicated a clustered pattern of wasted and severely children (***Figures 2*** and ***3***).

**Figure 4:**
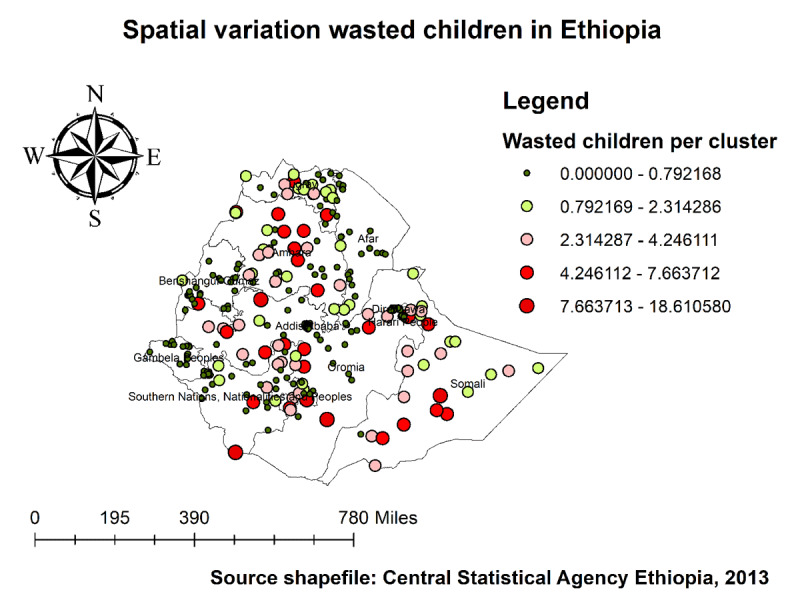
Spatial distribution of wasting among under-five children in Ethiopia, 2019 EDHS.

**Figure 5:**
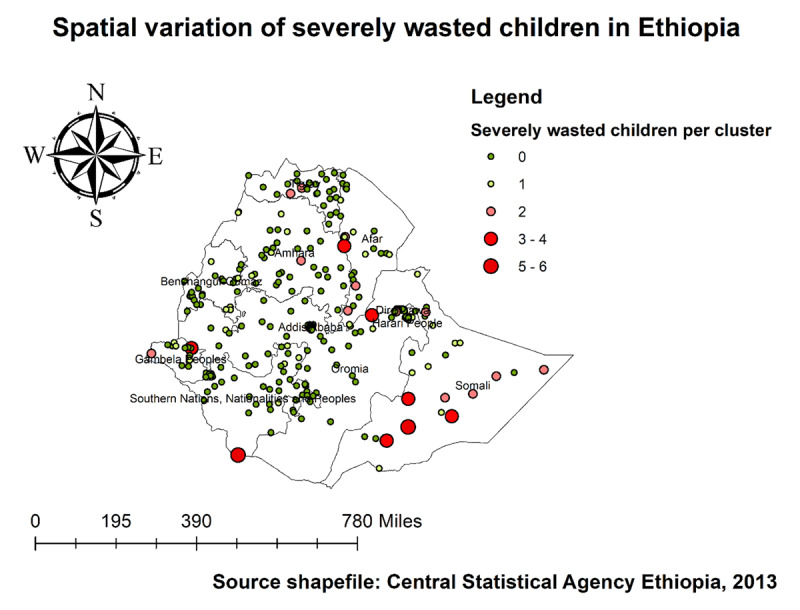
Spatial distribution of severely wasted under-five children in Ethiopia, 2019 EDHS.

**Figure 6:**
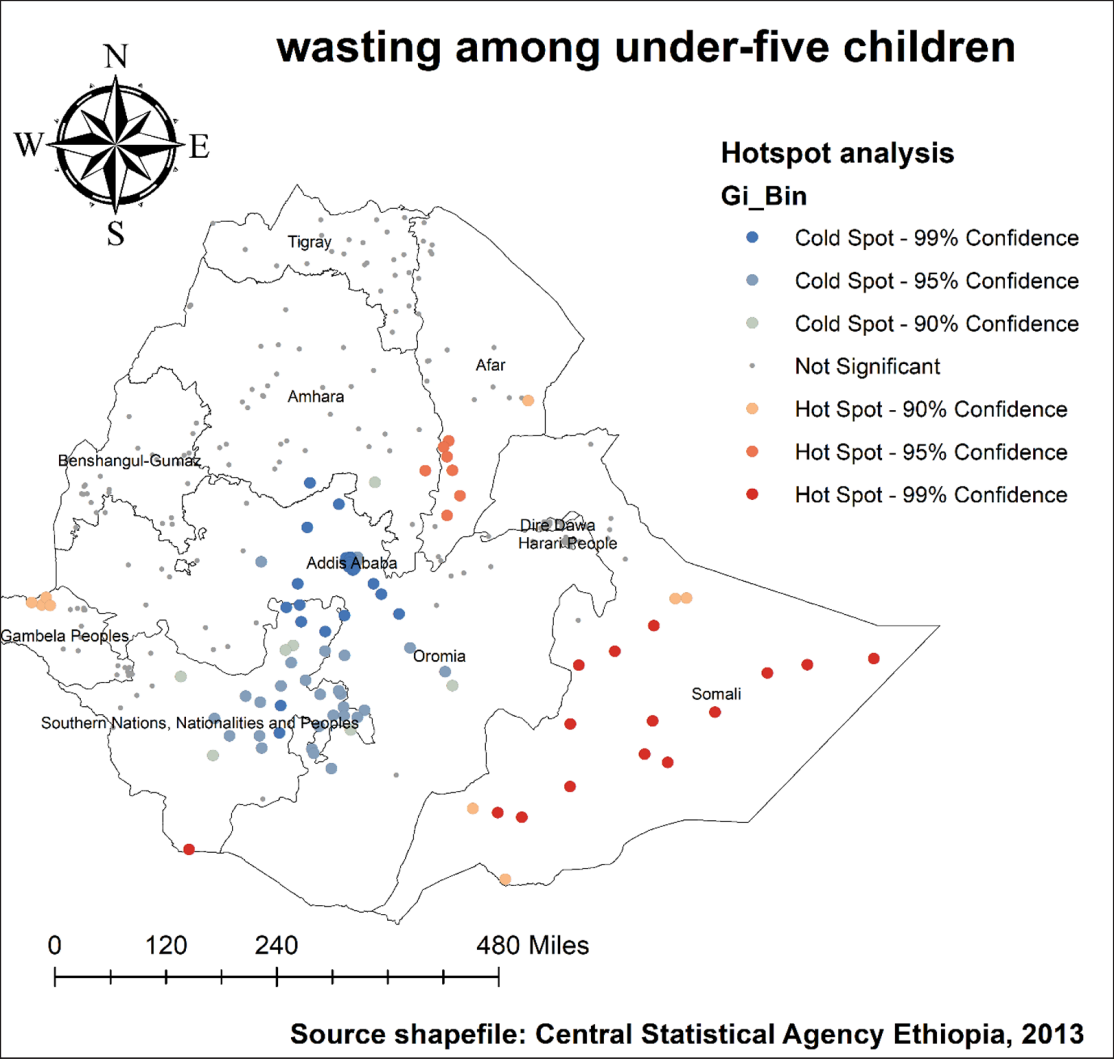
Hotspot analysis of wasting among under-five children in Ethiopia, 2019 EDHS.

**Figure 7:**
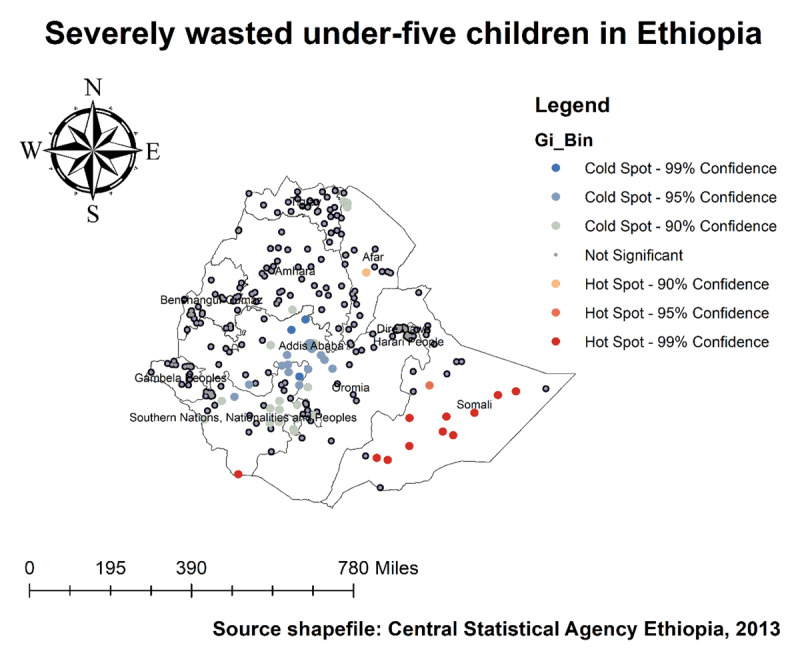
Hotspot analysis of severely wasted under-five children in Ethiopia, 2019 EDHS.

As we see in ***Figure 4*** and ***5***, The highest number of wasted and severely wasted children was observed in the Somali, Afar, and South Omo of the SNNP region. Further, ***Figure 6*** shows the hotspot areas of wasting among under-five children in Ethiopia. In the 2019 EDHS, the highest prevalence of wasted children (hotspots) was identified in Somali, Afar, SNNP, and Gambella regions. We observed that the prevalence of wasting is worse in the Somali region of Ethiopia. In regard to severely wasted children, the local (Getis-Ord Gi*) statistics indicated that Somali and South Omo of SNNP was identified as hotspot areas; whereas Addis Ababa and the central part of Oromia were identified as cold spots region of the country (***Figure 7***).

#### Kriging Interpolation of Wasting Among Children

**Figure 8:**
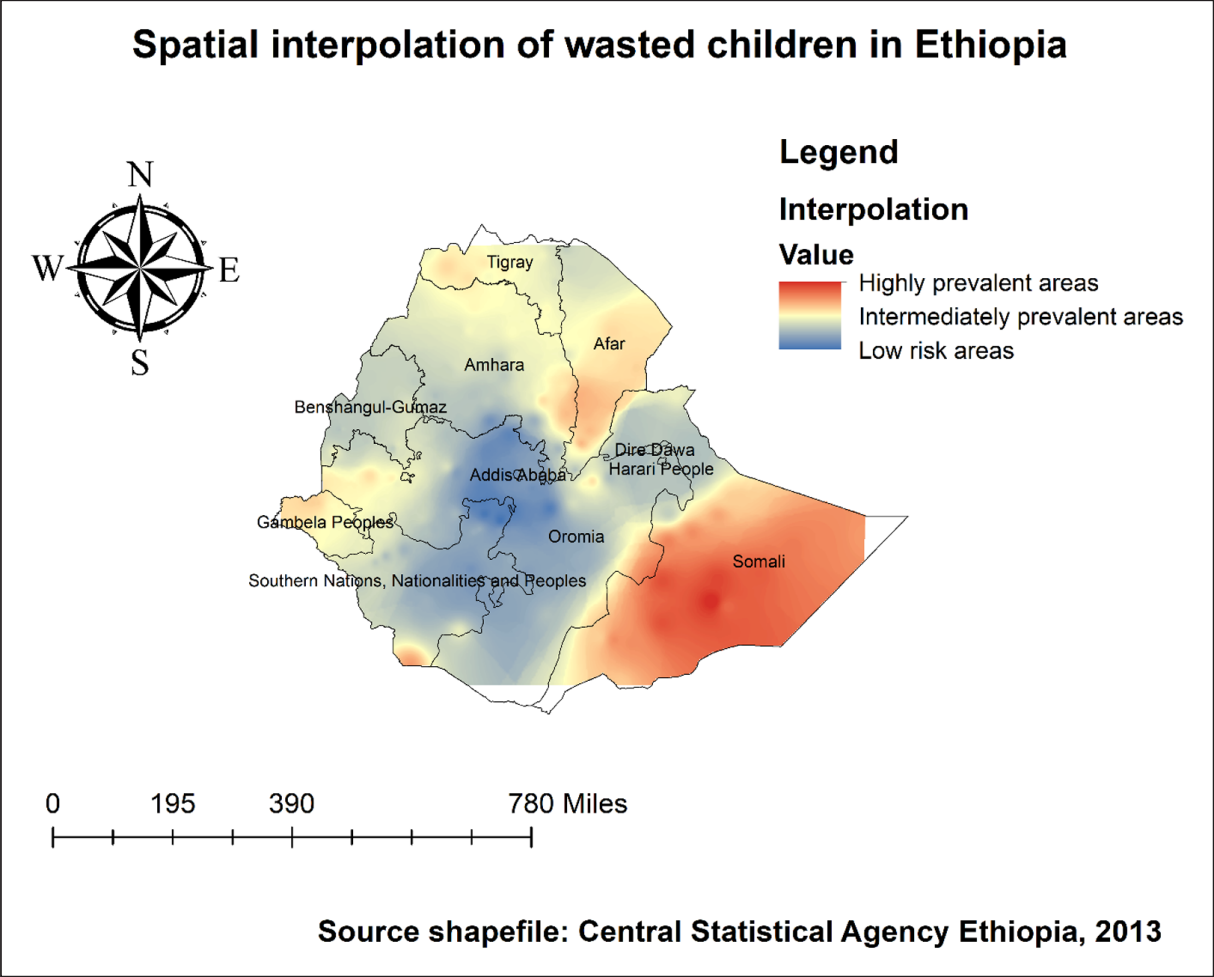
Spatial Interpolation of wasting among under-five children in Ethiopia, EDHS 2019.

**Figure 9:**
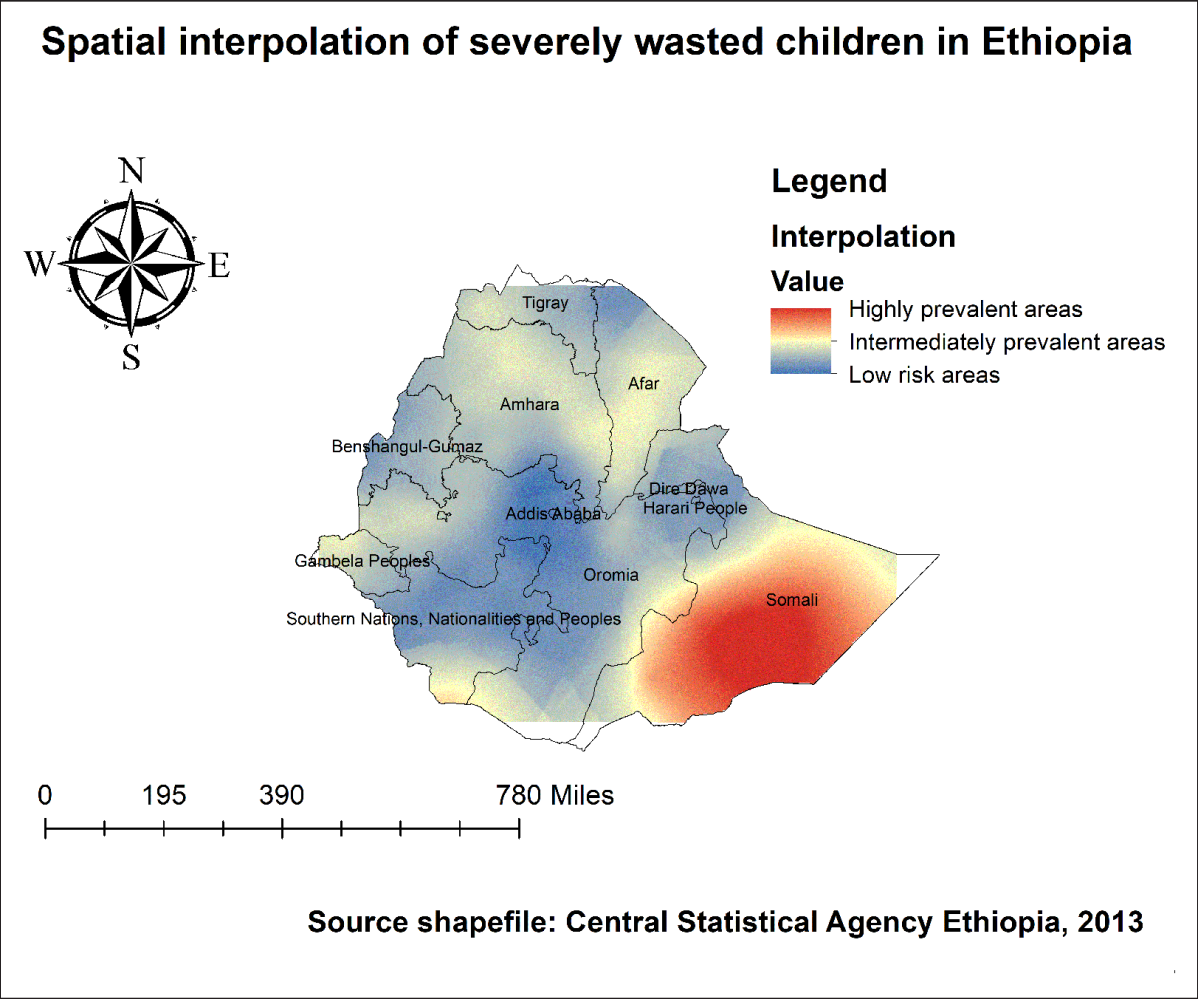
Spatial Interpolation of severely wasted children in Ethiopia, EDHS 2019.

The kriging interpolation analysis mapped the estimated distributions of wasting interpolating the available data to the areas where data were not collected. The red prediction areas show predicted prevalent areas of wasting among children. Based on EDHS 2019, Kriging interpolation predict that wasted children were detected in the Somali, Afar, border areas of Tigray, South Omo of SNNPR, and border areas of Gambella regions (***Figure 8***). Furthermore, Somali and SNNP (South Omo) areas were predicted as more risky areas for Severe malnutrition among children compared to other regions (***Figure 9***).

#### Spatial Scan Statistical Analysis

**Figure 10:**
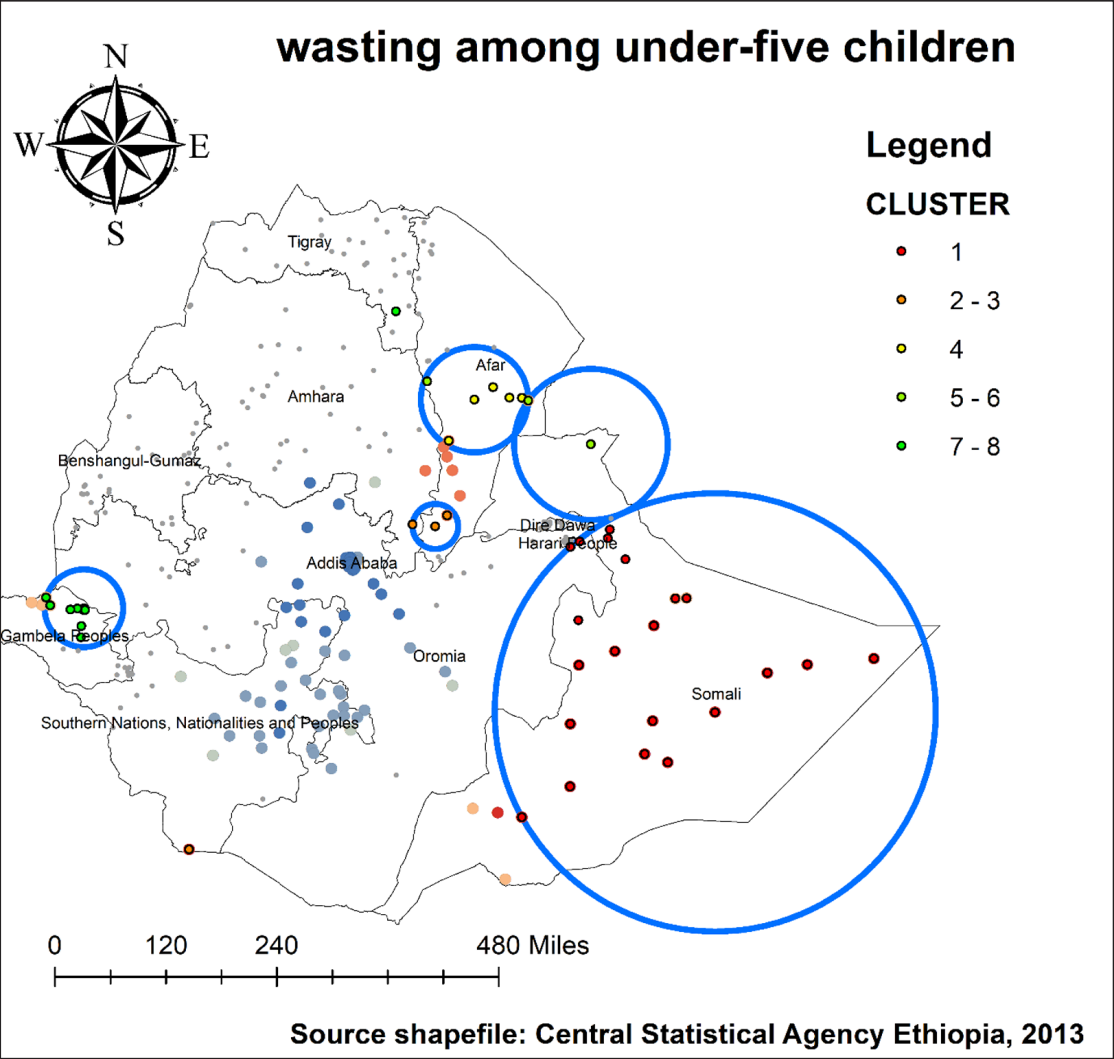
SaTScan scan analysis of wasting among under-five children in Ethiopia, 2019 EDHS.

In 2019 EDHS, a total of 25 significant clusters with wasted and 13 clusters with severely wasted children were identified. Of which, 21 and 12 of them were most likely (primary) clusters, respectively. Both spatial windows were located in the Somali region of Ethiopia. The spatial window for wasted children was centered at 639662 N, 44.465853 E with 381.04 km radius, with a relative risk (RR) of 2.9 and Log-Likelihood ratio (LLR) of 46.96, at p < 0.000. This means children within the spatial window had 2.9 times more wasted than children outside the window (***Table 2, Figure 10***).

**Table 2:** Significant Spatial Clusters of Wasting Among Under-Five Children in Ethiopia, 2019.


YEAR	CLUSTER	ENUMERATION AREAS (CLUSTERS DETECTED)	COORDINATES/RADIUS	POPULATION	CASES	RR	LLR	P-VALUE

**2019**	**1**	135, 123, 140, 137, 138, 124, 131, 145, 132, 122, 134, 136, 142, 133, 139, 129, 121, 130, 107, 250, 141	6.639662 N, 44.465853 E/381.04 km	515	116	2.90	46.96	**<0.000**

	**2**	193	4.495034 N, 36.230625 E/0 km	36	20	6.25	24.95	**<0.000**

	**3**	42, 40, 69	9.548779 N, 40.084216 E/39.15 km	60	20	3.75	13.72	**<0.000**


** N.B LLR, log-likelihood ratio; RR, relative risk.

**Figure 11:**
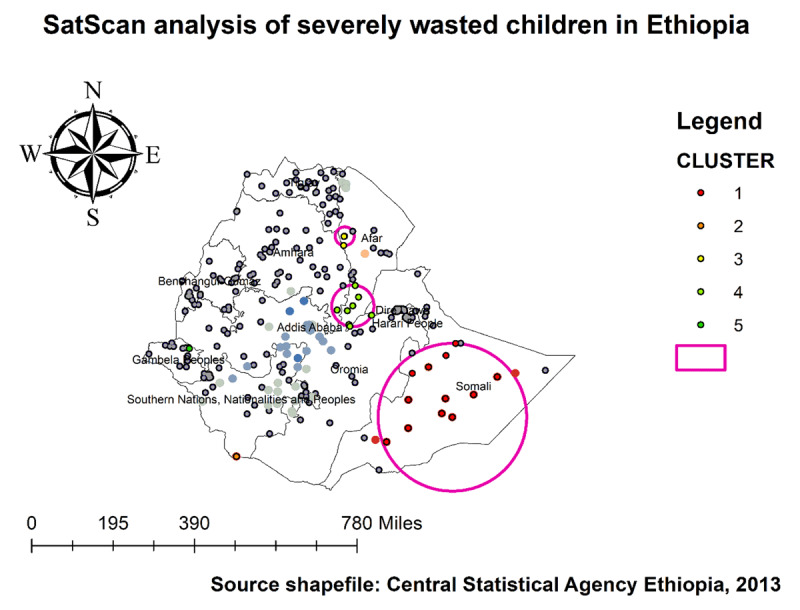
SaTScan scan analysis of severely wasted children in Ethiopia, 2019 EDHS.

Furthermore, the severely wasted clusters’ spatial window was centered at 5.856584 N, 43.726016 E with 284.96 km radius, with a RR of 6.4 and LLR of 24.2, at p < 0.001 (***Table 3***, ***Figure 11***).

**Table 3:** Significant Spatial Clusters Severely Wasted Children in Ethiopia, 2019.


YEAR	CLUSTER	ENUMERATION AREAS (CLUSTERS DETECTED)	COORDINATES/RADIUS	POPULATION	CASES	RR	LLR	P-VALUE

**2019**	**1**	137, 138, 123, 135, 142, 136, 145, 134, 140, 131, 141, 122	5.856584 N, 43.726016 E/284.96 km	291	26	6.4	24.2	**<0.001**

	**2**	193	4.495034 N, 36.230625 E/0 km	36	6	9.8	8.56	**<0.030**


** N.B LLR, log-likelihood ratio; RR, relative risk.

### Multilevel Analysis

From the total variation in acute malnutrition across the participants, 61% in 2019 EDHS was attributable to clustering. The clustering effect shown up here directed us to take multilevel analyses (***Table 4***).

**Table 4:** Model estimates for factors associated with acute malnutrition in 2019.


2019 EDHS	MODEL 0	MODEL 1	MODEL 2	MODEL 3

Inter-cluster correlation (ICC)	0.61	0.22	0.11	0.04

Log-likelihood ratio (LLR)	–1580.1	–1014.7	–1480.7	–984.0

Proportional change in variance (PCV)	Reference	0.61	0.71	0.72


The result of the multilevel analysis is presented below, adjusted odds ratios (AOR) for 2016 EDHS are shown in ***Table 4***. In multivariable multilevel mixed-effect logistic regression analysis at the individual/household level, the odd of being wasted was more than two times higher among male children, compared to females. Similarly, children from the poorest households had a greater odd of being wasted as compared to children from the richest households. Furthermore, among community-level factors, children living in Oromiya, Harari, and the Addis Abeba regions were associated with having a lower odd of being wasted compared to Tigray. Conversely, children from the Somali region were about 2.7 times more likely to be wasted (AOR = 2.7, 95% CI = 1.7–4.3) as compared to children from Tigray.

After fitting the mixed effect model, the odds of being wasted were higher among male children with an AOR of 1.9 [1.3–2.7]. The odds of being wasted were lower in children who are older with an AOR of 0.5[0.2–0.9]. Compared to Tigray region, respondents from Oromia, Benishangul, SNNP, Harari, Addis Ababa, and Dire Dawa regions had lower odds of wasted children’s with AOR of 0.3 [0.1–0.6], 0.3 [0.2–0.7], 0.4 [0.2–0.7], 0.3 [0.2–0.8], 0.2 [0.1–0.8], and 0.4 [0.2–0.5], respectively (***Table 5***).

**Table 5:** Factors Associated with Wasting Among Children in Ethiopia by Multilevel Logistic Regression Analysis, EDHS 2019.


VARIABLES	MODEL I	MODEL II	MODEL III

**Sex of child**			

Male	1.8 (1.3–2.7)***	—	1.9 (1.3–2.7)***

Female	1.00	—	1.00

**Child’s age(months)**			

0–5m	1.00	—	1.00

6–11 m	0.7 (0.4–1.3)	—	0.6 (0.3–1.2)

12–23 m	0.9 (0.6–1.7)	—	1.0 (0.5–1.8)

24–35m	0.8 (0.5–1.6)	—	0.9 (0.5–1.7)

>36m	0.5 (0.3–0.9)*	—	0.5 (0.2–0.9)*

**Wealth index**			

Poorest	1.00		1.00

Poorer	0.8 (0.5–1.2)	—	1.1 (0.6–1.8)

Middle	0.6 (0.3–1.1)	—	0.8 (0.4–1.5)

Richer	0.6 (0.4–1.1)	—	0.9 (0.5–1.7)

Richest	0.5 (0.2–0.8)*	—	0.6 (0.3–1.4)

**Source of drinking water**			

unimproved	1.4 (0.9–2.2)		1.5 (1.0–2.3)*

improved	1.00		1.00

**Region**			

Tigray	—	1.00	1.00

Afar	—	1.6 (0.9–2.8)	1.1 (0.5–2.1)

Amhara	—	0.8 (0.5–1.4)	0.7 (0.4–1.3)

Oromiya	—	0.5 (0.3–0.8)*	0.3 (0.1–0.6)***

Somali	—	2.7 (1.7–4.3)***	1.5 (0.7–2.9)

Benishangul Gumz	—	0.7 (0.3–1.3)	0.3 (0.2–0.7)***

SNNP	—	0.6 (0.4–1.3)	0.4 (0.2–0.7)***

Gambella	—	1.5 (0.9–2.7)	1.4 (0.7–2.9)

Harari	—	0.4 (0.2–0.8)*	0.3 (0.2–0.8)*

Addis Abeba	—	0.3 (0.1–0.7)*	0.2 (0.1–0.8)*

Dire Dawa	—	0.7 (0.3–1.2)	0.4 (0.2–0.9)*


## Discussion

Malnutrition is the most sensitive health indicator that reflects the quality of the health care delivery system and socio-economic progress of a country [1, 9]. Acute malnutrition or wasting is a health indicator and a critical measure of children’s nutritional status [17]. We examined the nutritional status of children aged 6–59 months in Ethiopia through a spatial and multilevel analysis approach based on the 2019 Ethiopian Demographic and Health Survey. This study revealed that 7% of children in Ethiopia had acute malnutrition in the 2019 national survey. The current magnitude of wasted children is also higher than the national target of 3% [17]. There are more than a few factors associated with this at both individual and contextual levels. The sex of a child, age of the child, household wealth status, source of water, and region of residence are amongst those predictors; however, family size, vaccination status, place of residence, respondent ANC status, and birth order of the child not deemed significant here.

This study showed that the household wealth index was a significant predictor of children’s nutrition status, Children from the poorest household wealth index had higher odds of being wasted than children from the richest households. This finding is supported by previous studies conducted in Ghana [23], Uganda [7], Bangladesh [30], Pakistan [32], and Ethiopia [24,26,27]. This might be due to the better household wealth status associated with their ability to improve nutritional choices, high access to health information, attitude change, and address basic nutritional and hygiene behaviors that may prevent nutritional failure. Further, among the main finding of this study, male children had a satisfied association with being wasted more than females. Similar findings have been reported by other studies [7,30].

The spatial autocorrelation analysis result indicated that acute malnutrition and severe acute malnutrition had a spatial dependency in the 2019 EDHS (Moran’s I: 0.21 and 0.11, respectively at p-value 0.01). This result is supported by the findings in Somalia [39], Myanmar [36], and WHO [40], and Global Burden of Disease data [41]. Spatial analysis portrayed that under-five children in the pastoralists region were at higher risk of acute malnutrition compared to other regions; however, acute malnutrition or wasting was also found severe in some pocket areas of SNNP and Gambella. Previous studies were in line with a higher level of wasted children in the pastoralists regions [27,39,42]. The Afar and Somali regions might be always at risk because of the poor access to healthcare services and feeding practices depending on their way of life. The finding of both spatial and statistical analyses (***Table 5***), identified high-risk regions consistently.

These findings have valuable policy implications for intervention and program design. The hot spot areas of acute and severe malnutrition can be detected at local administrative levels. Generally, these findings are supremely important for the Ministry of Health and Regional Health Bureau to give attention to those hot spot areas to have good progress towards achieving sustainable development goal targets for nutrition and under-five mortality and morbidity [2,16].

As a strength, the study used data from a nationally representative population-based study with a high response rate, which results in give high statistical power to infer the characteristics of the study population. Also, the sampling weight was applied to produce reliable estimates. Another important strength of this study is the use of multilevel logistic regression and spatial analysis, which was able to cross-validation of results. However, it has the following limitations. The cross-sectional nature of the study prevents causality from being inferred between the independent and dependent variables. Furthermore, this study did not consider spatial covariates that may allow discovering more spatial and temporal structures, which were not investigated as part of the survey.

## Conclusion

Overall, the acute malnutrition among under-five children in Ethiopia has remained a public health problem and the distribution of risk was followed the contextual nature of the regions. The hotspot (high risk) areas of both acute and severe malnutrition were detected in the Somali and Afar regions. Moreover, border areas of Gambela and the south Omo zone of SNNP regions were at higher acute malnutrition. This spatial distribution might be very fundamental to develop strategies and localized interventions. Similarly, in multilevel analysis both individual and community-level factors were significantly associated with acute malnutrition among children aged 6–59 months. Predictors such as the household living standards and wealth index were consistently related to improved children nutritional status.

Therefore, the fact that the spatial distribution and statistical association were well supported by one another showed the distribution is scientifically sound. As a practical recommendation, the authors believe that public health intervention activities designed in a targeted approach to impact high-risk populations as well as geographic regions were vital to narrow acute malnutrition in Ethiopia.

## Data availability statement

Data we used for this study are publicly available in the MEASURE DHS program and you can access it from *www.measuredhs.com* after explaining the objectives of the study. Then after receiving the authorization letter, the data is accessible and freely downloaded.

## Abbreviations

AOR-Adjusted Odds Ratio, ANC- Antenatal Care, CSA-Central Statics Agency, CI-Confidence Interval, CIAF-Composite index of anthropometric failure, COR-Crude Odds Ratio, EDHS-Ethiopia Demographic and Health Survey, ICC-Intra Class Correlation Coefficient, LLR-Log-Likelihood Ratio, MOR-Median odds ratio, OR-Odds Ratio, PVC-Proportional Change in Variance, RR-Relative Risk, SNNPR-Southern Nations, Nationalities, and Peoples’ Region, WHO-World Health Organization.
